# Enteral versus intravenous approach for the sedation of critically ill patients: a randomized and controlled trial

**DOI:** 10.1186/s13054-018-2280-x

**Published:** 2019-01-07

**Authors:** Giovanni Mistraletti, Michele Umbrello, Silvia Salini, Paolo Cadringher, Paolo Formenti, Davide Chiumello, Cristina Villa, Riccarda Russo, Silvia Francesconi, Federico Valdambrini, Giacomo Bellani, Alessandra Palo, Francesca Riccardi, Enrica Ferretti, Maurilio Festa, Anna Maria Gado, Martina Taverna, Cristina Pinna, Alessandro Barbiero, Pier Alda Ferrari, Gaetano Iapichino, the SedaEN investigators, Gaetano Iapichino, Alberto Morabito, Martin Langer, Franco Valenza, Roberto Malacrida, Marco Rambaldi, Giovanni Mistraletti, Davide Chiumello, Michele Umbrello, Paolo Formenti, Paolo Spanu, Stefania Anania, Elisa Andrighi, Alessandra Di Carlo, Federica Martinetti, Serena Barello, Andrea Noto, Gianfranco Capello, Bruno Sabatelli, Giovanni Brenna, Morena Astori, Pietro Placido, Luciano Gattinoni, Alessandro Protti, Paolo Cadringher, Riccarda Russo, Francesca Pagan, Virna Berto, Paola Roselli, Giulio Ronzoni, Eduardo Beck, Silvia Francesconi, Maurizio Gaiotto, Danilo Radrizzani, Luca Ferla, Federico Valdambrini, Riccardo Giudici, Laura Merlini, Antonio Pesenti, Giacomo Bellani, Alessia La Bruna, Emanuele Rezoagli, Alberto Lucchini, Antonio Braschi, Alessandra Palo, Thekla Niebel, Marina Selvini, Sergio Cortesi, Attilio Quaini, Giorgio Iotti, Francesca Riccardi, Enrico Contri, Antonella Sacchi, Sergio Livigni, Giuseppe Naretto, Enrica Ferretti, Alessandro Deprado, Virna Venturi degli Esposti, Pietro Caironi, Giulio Radeschi, Maurilio Festa, Lorenzo Odetto, Daniele Ferrero, Stefano Cognolato, Roberto Penso, Roberta Vacchelli, Silvano Cardellino, Edda Bosco, Anna Maria Gado, Anna Bresciani, Ivana Pozzo, Annachiara Alessio, Vanessa Clarindo Rodrigues, Edna Biase, Nicoletta Vivaldi, Martina Taverna, Antonella Nava, Cristina Pinna, Francesco Ponzetta, Lucilla Bavutti, Paola Martina, Beatriz Palacios, Giancarla Bergonzini

**Affiliations:** 1 Dipartimento di Fisiopatologia Medico-Chirurgica e dei Trapianti, Università degli Studi di Milano, A.O. San Paolo - Polo Universitario, Via A. Di Rudinì, 8, 20142 Milano, Italy; 2 SC Anestesia e Rianimazione, ASST Santi Paolo e Carlo, Ospedale San Paolo - Polo Universitario, Milano, Italy; 3 Dipartimento di Economia, Management e Metodi Quantitativi, Università degli Studi di Milano, Milano, Italy; 4 Dipartimento Anestesia, Rianimazione ed Emergenza-Urgenza, Fondazione IRCCS Ca’Granda, Ospedale Maggiore Policlinico, Milano, Italy; 5 Dipartimento di Scienze della Salute, Università degli Studi di Milano, Milano, Italy; 6 UOC Anestesia e Rianimazione, ASST Monza, Ospedale di Desio, Monza, Italy; 7 UO Anestesia e Rianimazione, ASST Ovest Milanese, Ospedale Nuovo di Legnano (MI), Legnano, Italy; 8 Dipartimento di Medicina e Chirurgia, Università degli Studi Milano Bicocca, A.O. San Gerardo, Monza, Italy; 9 Dipartimento Medicina Intensiva, IRCCS Fondazione Policlinico San Matteo, Pavia, Italy; 10 UO Anestesia e Rianimazione 2, IRCCS San Matteo, Pavia, Italy; 11 SC Anestesia Rianimazione B DEA, Ospedale San Giovanni Bosco, Torino, Italy; 12 SCDU Anestesia e Rianimazione, AOU San Luigi Gonzaga di Orbassano (TO), Torino, Italy; 13 UO Anestesia e Rianimazione, AO Cardinal Massaia, Asti, Italy; 14 UO Anestesia e Rianimazione, AO Santi Antonio e Biagio e Cesare Arrigo, Alessandria, Italy; 15 UO Anestesia e Rianimazione, Nuovo Ospedale Civile Sant’Agostino Estense, Modena, Italy

**Keywords:** Hypnotics and sedatives, Hydroxyzine, Melatonin, Patient care planning, Nursing education research

## Abstract

**Background:**

ICU patients must be kept conscious, calm, and cooperative even during the critical phases of illness. Enteral administration of sedative drugs might avoid over sedation, and would be as adequate as intravenous administration in patients who are awake, with fewer side effects and lower costs. This study compares two sedation strategies, for early achievement and maintenance of the target light sedation.

**Methods:**

This was a multicenter, single-blind, randomized and controlled trial carried out in 12 Italian ICUs, involving patients with expected mechanical ventilation duration > 72 h at ICU admission and predicted mortality > 12% (Simplified Acute Physiology Score II > 32 points) during the first 24 h on ICU. Patients were randomly assigned to receive intravenous (midazolam, propofol) or enteral (hydroxyzine, lorazepam, and melatonin) sedation. The primary outcome was percentage of work shifts with the patient having an observed Richmond Agitation-Sedation Scale (RASS) = target RASS ±1. Secondary outcomes were feasibility, delirium-free and coma-free days, costs of drugs, length of ICU and hospital stay, and ICU, hospital, and one-year mortality.

**Results:**

There were 348 patients enrolled. There were no differences in the primary outcome: enteral 89.8% (74.1–100), intravenous 94.4% (78–100), *p* = 0.20. Enteral-treated patients had more protocol violations: *n* = 81 (46.6%) vs 7 (4.2%), *p* < 0.01; more self-extubations: *n* = 14 (8.1%) vs 4 (2.4%), *p* = 0.03; a lighter sedative target (RASS = 0): 93% (71–100) vs 83% (61–100), *p* < 0.01; and lower total drug costs: 2.39 (0.75–9.78) vs 4.15 (1.20–20.19) €/day with mechanical ventilation (*p* = 0.01).

**Conclusions:**

Although enteral sedation of critically ill patients is cheaper and permits a lighter sedation target, it is not superior to intravenous sedation for reaching the RASS target.

**Trial registration:**

ClinicalTrials.gov, NCT01360346. Registered on 25 March 2011.

**Electronic supplementary material:**

The online version of this article (10.1186/s13054-018-2280-x) contains supplementary material, which is available to authorized users.

## Introduction

The management of pain, agitation, and delirium is a key point in the care of critically ill patients [1]. Once triggering conditions have been dealt with, pharmacological treatment becomes necessary. After adequate analgesia, sedative drugs, usually given by continuous intravenous (IV) infusion, ensure comfort and allow life-saving procedures, constituting an invaluable tool during the ICU stay. However, they have several side effects [2, 3]. International guidelines [4, 5] suggest using the lowest effective doses for early achievement [6, 7] and constant maintenance of a light level of sedation even in the most severe conditions [8, 9]. Several strategies have been proposed to avoid deeper-than-needed [10] levels of sedation, aiming for the goal of keeping ICU patients “calm, conscious, and cooperative” [11–13].

However, even if unjustified [8], a large proportion of ICU staff still tend to consider this unfeasible [14] because of the risk of self-removal of invasive devices [15], the fear of greater stress/discomfort among patients, and the increased workload for operators. Despite the widespread use of validated scoring systems for sedation, like the Richmond Agitation and Sedation Scale (RASS) [16], a very large proportion of ICU patients are kept at a sedation level deeper than desired [10, 17], quite likely causing avoidable side effects.

With continuous intravenous (IV) infusion one can predict the duration of the drug effect using pharmacokinetic calculations. This approach, safe for patients with a short ICU stay, could be useless or dangerous in patients needing mechanical ventilation (MV) for more than 3 days. In these cases, it may cause over-administration [18] even with adequate sedation targets. Moreover, the daily awakening trials [15] could induce non-physiological neurological fluctuations, preventing the formation of factual memories, and becoming a precipitant cause of delirium [19], leading to post-ICU cognitive dysfunction [20].

Analgesic [21] and sedative [22] drugs are rarely administered enterally (EN) because of their slower onset of effect and unpredictable pharmacokinetics, even when intestinal absorption is adequate from ICU admission [23]. Our “EN sedation” protocol [24] uses hydroxyzine (a first-generation antihistaminic drug, with antiemetic and gastric antisecretory properties) and allows the addition of low doses of lorazepam (a medium-half-life benzodiazepine) if necessary. Melatonin is continuously used [25] as a physiological sleep-inducer, with antioxidant, anti-inflammatory, analgesic, and immune-modulating properties [26, 27].

The longer onset and offset time of EN administration make this approach difficult. However, at the same time, this route ensures a more stable level of consciousness with less neurological fluctuation and fewer cardiorespiratory side effects. EN drugs cost much less than IV ones [22], are less likely to lead to deep sedation, and are similarly effective as judged by nurses, if an awake target is desired [12].

The hypothesis of the present study is that an unusual EN sedative drugs administration protocol could reach and maintain light and effective sedation, compared to the more common IV continuous infusion. The main outcome was achievement of the target sedation level in ICU patients needing MV for more than 72 h.

## Methods

### Study design

The methods have been described in detail elsewhere [24]. Briefly, the “Enteral versus intravenous sedation trial (SedaEN)” is a randomized, controlled, multicenter, single-blind trial (ClinicalTrials.gov, NCT01360346), to compare two protocols for sedation management, both used after adequate analgesia. In the control group, propofol or midazolam were given by IV infusion. In the intervention group, melatonin, hydroxyzine, and possibly lorazepam were administered enterally, while IV drugs were allowed during the first 48 h on ICU [25].

### Setting

The 12 Italian participating ICUs were selected on the basis of their availability to use two very different protocols simultaneously; in order to obtain the best generalizability, they were heterogeneous in terms of patient case-mix, central/rural area, previous knowledge about EN sedation, and belonging to academic hospitals. To help in caring for complex cases, three flowcharts were proposed, for pain, agitation, and delirium management [24]. Weaning from MV was not set in the protocol, but was managed according to local guidelines in both groups.

### Participants

**Fig. 1:**
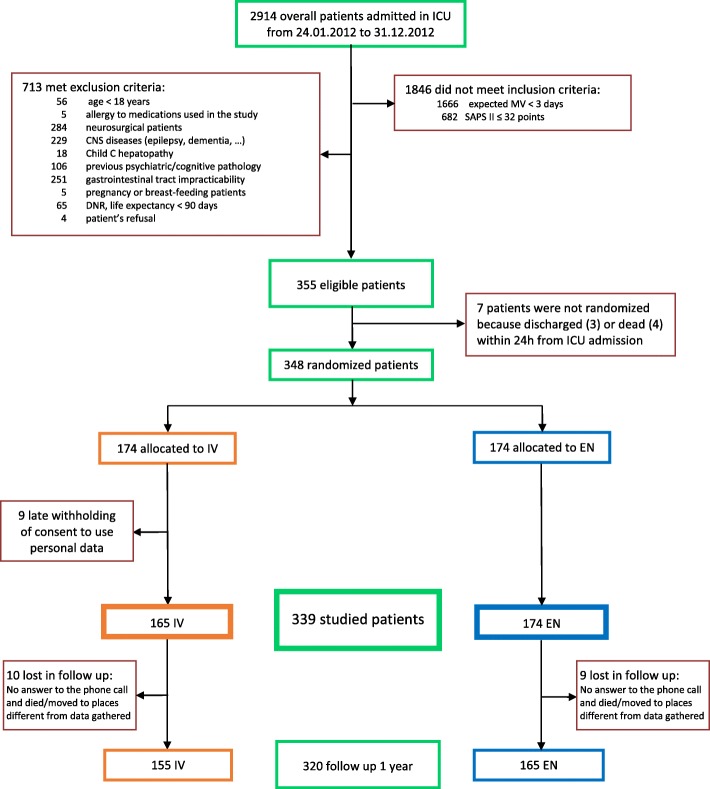
The Consolidated Standards of Reporting Trials (CONSORT) diagram for screening and randomization in the “Enteral versus intravenous sedation” (SedaEN) trial. The 339 patients were analyzed using an intention-to-treat approach, without considering treatment interruptions. ICU, intensive care unit; MV, mechanical ventilation; SAPS II, Simplified Acute Physiology Score II; CNS, central nervous system; DNR, do not resuscitate orders; IV, intravenous; EN, enteral

Inclusion criteria were age ≥ 18 years, MV duration > 72 h as estimated by the physician in charge at ICU admission, and Simplified Acute Physiology Score II (SAPS II) [28] > 32 points during the first 24 h on ICU, corresponding to expected mortality > 12%. Exclusion criteria are set out in Fig. 1. A study sample of 300 patients was calculated as required in order to detect a clinically relevant difference in the main outcome [24].

### Randomization and masking

Patients were randomized through a centralized website [24]. After written informed consent had been obtained from patients or relatives according to the indications of the 12 local ethics committees that approved the study, the group allocation was established with a minimization algorithm balanced within centers. Once a patient was assigned, no change of protocol arm was allowed. Staff members were aware of the group assignment, as it was not possible to blind staff to the sedative drug administration route.

### Procedures

According to a patient-centered approach [1, 4, 5], in both groups the attending physicians were invited to state the target sedation level for each work shift, aiming as soon as possible for a conscious, calm, and cooperative target, and to titrate doses of sedatives early with the rule of “timing, adequacy, de-escalation”. Physicians on duty discussed prescriptions at least twice a day during handovers. Nurses assessed the depth of sedation, indicating the prevalent RASS level in their work shift, and stated whether the prescriptions were adequate for the severity of the illness, the invasive procedures, and patients’ surveillance and security. Even though protocol violations were strongly discouraged, they were always allowed and recorded. IV boluses of analgesics (fentanyl or morphine) and/or IV sedatives (propofol or midazolam) were not considered violations in the EN arm when used for extemporary invasive/painful/surgical procedures.

### Outcomes

The main outcome was the percentage of work shifts in which the desired sedation level was reached or nearly reached (observed RASS = target RASS ±1). The secondary outcomes were feasibility of the sedation protocol (percentage of shifts with assigned protocol violations); delirium-free and coma-free days, assessed by the Confusion Assessment Method for ICU (CAM-ICU) [29] and RASS (coma was defined by RASS levels of − 4 and − 5); ventilation-free days; nursing assessment of the adequacy of sedation (anxiety, cooperation, tolerance of the environment); length of ICU stay; ICU, hospital, and one-year mortality; hospital costs for neuroactive drugs [30]. Adverse events such as self-extubation and removal of other invasive tools, unscheduled diagnostic neurological tests, anxiety, hours of sleep and agitation, and use of anti-psychotics, pharmacological antagonists, or physical restraints were recorded.

### Statistical analysis

An intention-to-treat statistical approach was planned, because the violation rate was unpredictable a priori. Baseline patient characteristics and single-observation outcomes were analyzed by two-tailed tests: the Wilcoxon rank-sum test for analysis of continuous data and Fisher’s exact test for analysis of categorical data. We performed repeated measures analysis for data recorded during the whole ICU stay; comparisons were made by multilevel mixed-effects Poisson regression. This statistical approach was selected to simultaneously analyze the net effects of group assignment, the effect of time spent in ICU, and the cumulative effect of the sedatives, as calculated by multiplying the group (EN = 1, IV = 0) and the ICU day from group assignment, to highlight the adjunctive effects of the daily EN doses of sedatives.

Mortality was analyzed by log-rank test and presented as Kaplan–Meier curves, without adjustment for baseline covariates. There were no missing data on the main outcome, as the centralized website needed these data to be completed before allowing the validation of each patient’s recordings. The Stata 12 statistical package (Stata Corporation, College Station TX, USA) was used for all statistical analyses.

After the first 140 patients were enrolled, an interim analysis was planned. The results were discussed in a steering committee meeting (28 May 2012). In the power calculation, a study sample of 141 patients per group (power 80%, alpha 0.05) was calculated as sufficient to observe a 15% difference in the prevalence of sedation adequacy (observed RASS = target RASS ±1) between the two study arms: such a difference was considered clinically relevant and likely to influence medical practice. To allow for missing data, a total of 300 patients was expected to be enrolled, with at least 20 patients per ICU [24].

## Results

### Participants

The characteristics of the 12 participating ICUs are described in Additional file 1: Table E1. During the study (24 January 2012 to 31 December 2012), 2914 critically ill patients were admitted and screened; 348 of them were randomized (Fig. 1). The baseline characteristics of patients at ICU admission are presented in Table 1; the two groups were adequately balanced according both the criteria stated a priori and in the other clinical parameters.

**Table 1:** Baseline characteristics

	Group IV (*N* = 165)	Group EN (*N* = 174)
Age, median [IQR], years^a^	71 [62–77]	73 [62–78]
Men^a^	107 (64.8)	109 (62.6)
BMI, median [IQR]	25.9 [23.7–29.4]	26.1 [23.4–29.4]
Severe sepsis or septic shock^a^	48 (29.1)	64 (36.8)
SAPS II score, median [IQR]^a, b^	45 [38–55]	46 [38–54]
SOFA score, median [IQR] ^c^	8 [5–10]	7 [5–10]
Type of admission^a, d^		
Medical	110 (66.7)	114 (65.5)
Surgical/traumatic	55 (33.3)	60 (34.5)
Admission from		
Emergency room	55 (33.3)	61 (37.0)
Ward	55 (33.3)	59 (35.8)
Operating theatre	39 (23.6)	34 (20.6)
Other ICU	16 (9.7)	20 (12.1)
Reason for ICU admission^e^		
Respiratory failure	97 (58.8)	101 (58.0)
Cardiac failure	38 (23.0)	40 (23.0)
Neurologic failure	11 (6.7)	12 (6.9)
Monitoring	6 (3.6)	9 (5.2)
Other	13 (7.9)	12 (6.9)
Acute or chronic kidney failure^a^	30 (18.2)	43 (24.7)
Moderate to severe COPD^a^	50 (30.3)	53 (30.5)

*Abbreviations: EN* enteral, *IV* intravenous, *BMI* body mass index, *IQR* interquartile range, *SAPS* Simplified Acute Physiology Score, *SOFA* Sequential Organ Failure Assessment, *COPD* chronic obstructive pulmonary disease, *ICU* Intensive Care Units

^a^Characteristics used by the minimization algorithm for the group assignment

^b^SAPS II may range from 0 to 163 points, with higher scores indicating more severe diseases

^c^SOFA score may range from 0 to 24 points, with higher scores indicating more severe diseases

^d^Surgical/trauma refers to admission from an operating room or postoperative recovery area

^e^Main reasons for admission are mutually exclusive

### Interim analysis

No serious adverse events were reported, and there was a significant difference in the RASS target: patients in the EN group were more frequently at a conscious level (RASS = 0) than those randomized to the IV group. Clear recommendations were communicated to all the local investigators during the two planned meetings (24–25 March and 15 September 2012), and during the principal investigator’s visits to each participating center. Even though they progressively decreased, these differences remained statistically significant until the study ended (82.9 vs 93.3%, *p* < 0.01).

### Outcomes

**Fig. 2:**
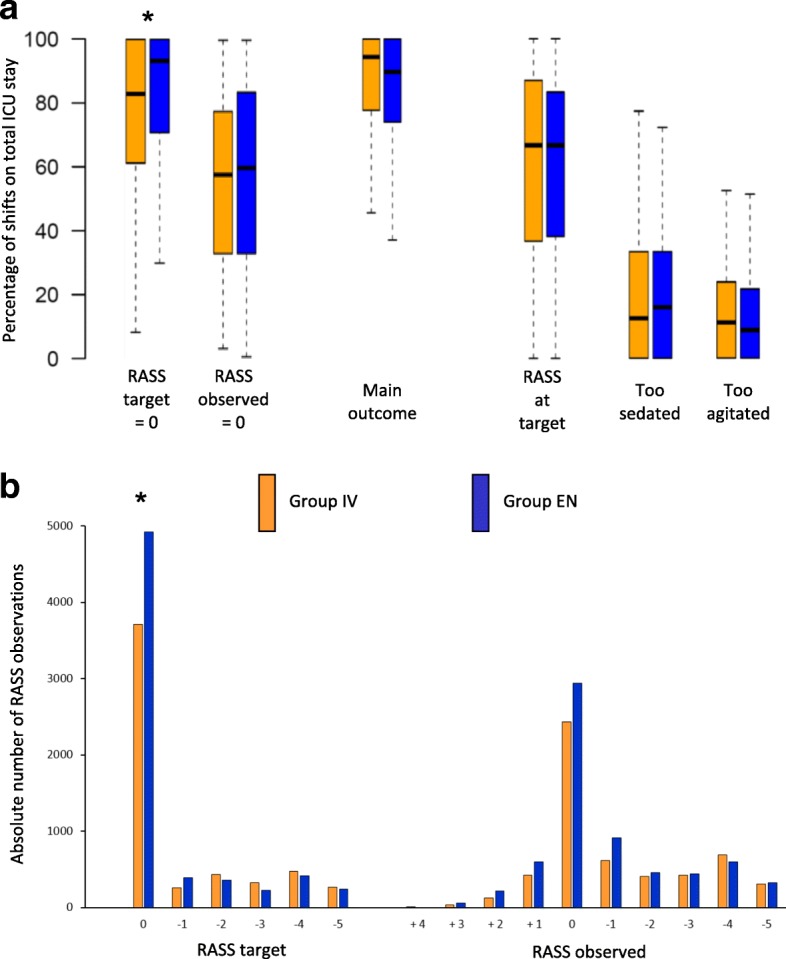
**A** Percentage of shifts in total ICU stay. Main outcome means the Richmond Agitation Sedation Scale RASS observed = RASS target ±1. RASS at target means RASS observed = RASS target. Too sedated means RASS observed < RASS target. Too agitated means RASS observed > RASS target. **B** Absolute number of RASS observations. *P <0.05. ICU, Intensive Care Unit; IV, intravenous; EN, enteral

The primary outcome of achieving the RASS target (Fig. 2a) was not different in the two groups (94.4 vs 89.8%, *p* = 0.20) (Table 2). Since the prevalence of RASS target = 0 was higher in the EN group, a multivariate generalized linear model was built to control for all the covariates: the effect of study group on the main outcome was confirmed as not significant (Additional file 1: Table E2).

**Table 2:** Study outcomes

	Group IV (*N* = 165)	Group EN (*N* = 174)	*P* value
Percentage of shifts at target RASS = 0, median [IQR]	82.9 [61.3–100]	93.3 [70.8–100]	< 0.01
Percentage of shifts at observed RASS = 0/− 1, median [IQR]	57.9 [33.3–77.8]	60.1 [33.3–83.7]	0.53
Main outcome			
Percentage of shifts at RASS observed = target ±1, median [IQR]	94.4 [77.8–100]	89.8 [74.1–100]	0.20
Secondary outcomes			
Percentage of adequate sedation, as judged by nurses, median [IQR]	92.4 [80.9–100]	89.7 [76.2–100]	0.11
Percentage of shifts with protocol violation, median [IQR]	0 [0–0]	0 [0–24.1]	< 0.01
Patients with protocol violation, *n* (%)	7 (4.2)	81 (46.6)	< 0.01
Coma-free days	27 [19–28]	27 [18–28]	0.80
Delirium-free days	27 [19–28]	27 [15–28]	0.40
Coma and delirium-free days	25 [11–28]	25 [10–28]	0.61
Ventilator-free days	21 [3–27]	22 [2–26]	0.89
Length of ICU stay	10 [6–18]	10 [6–18]	0.75
Mortality			
In ICU, *n* (%)	41 (24.8)	45 (25.9)	0.90
In hospital, *n* (%)	54 (32.7)	62 (35.6)	0.65
One year, *n* (%)	68 (43.9)	71 (43.0)	0.82
Daily cost for planned sedatives, €/ventday	1.64 [0.15–4.78]	0.38 [0.22–0.60]	< 0.01
Daily cost for unplanned sedatives, €/ventday	0 [0–0]	0.16 [0–2.15]	< 0.01
Daily cost for all neuroactive drugs, €/ventday	4.15 [1.20–20.19]	2.39 [0.75–9.78]	0.01
Self-removal of ET tube, *n* (%)	4 (2.4)	14 (8.1)	0.03
Need to replace ET tube, *n* (%)	3 (1.8)	10 (5.7)	0.09
Self-removal of other invasive tools, *n* (%)	21 (12.7)	29 (16.7)	0.36
Unscheduled neurological tests, *n* (%)	30 (18.2)	33 (19.0)	0.89

*Abbreviations*: *IV* intravenous, *EN* enteral, *RASS* Richmond Agitation Sedation Scale, *ICU* Intensive Care Unit, *ventday* day with mechanical ventilation, *ET* endotracheal

The prevalence of adequacy as judged by nurses was not different for the EN and IV groups (89.7% vs 92.4%, *p* = 0.11), but there were significantly more protocol violations in the EN group. The reported reasons for violations are presented in Additional file 1: Table E3. No differences were evident in coma-free, delirium-free, or ventilator-free days. Mortality did not differ between the groups either in the ICU (Additional file 1: Figure E1) or in hospital, or thereafter until one year after ICU discharge. There were more unplanned self-extubations in the EN group; however, there were no significant differences in the need to replace the endotracheal tube and none of these events were associated with death or other serious complications.

The drug doses and costs are presented in Additional file 1: Table E4. Daily doses of sedatives were very low overall and were similar to those in other studies [31]. Daily charges for planned sedatives were lower with the EN approach (1.64 vs 0.38 €/day, *p* < 0.01), but because unplanned sedatives were used more frequently, the sums of all daily charges for sedatives during MV did not differ between the groups (1.64 vs 0.74 €/day, *p* = 0.16). Considering all the neuroactive drugs used together (sedatives, analgesics, and antipsychotics), the total daily cost was significantly lower in the EN group (4.15 vs 2.39 €/day, *p* = 0.01).

Neurological observations were gathered during each staff shift, and are presented in Table 3. Patients in the EN group had higher RASS values, both for the target and for the actual value (Fig. 2b); the prevalence of coma was lower in this group, with no difference in the prevalence of delirium.

**Table 3:** Neurological observations

	Group IV *N* = 165	Group EN *N* = 174	*P* values		
Maximum number of theoretical observations	5529	6663	group	time	group*time
Pain (VNR ≥ 3 or BPS ≥ 6), *n* (%)	663 (12.0)	734 (11.0)	0.73	0.29	0.12
Anxiety (VNR > 0), *n* (%)	511 (33.7)	574 (32.4)	0.99	0.17	0.14
Physical restraint use > 1 h, *n* (%)	694 (18.0)	785 (16.2)	0.62	< 0.01	0.42
Sleep time > 2 h observed by nurses, *n* (%)	571 (86.1)	649 (86.3)	0.81	< 0.01	0.07
Agitation hours > 1, *n* (%)	311 (20.3)	304 (17.6)	0.81	0.46	0.51
Coma- and delirium-free shift, *n* (%)	3004 (64.5)	3552 (64.8)	0.20	< 0.01	< 0.01
Delirium (CAM-ICU ⊕), *n* (%)	644 (13.8)	998 (18.2)	0.72	0.02	0.98
Coma (RASS = − 4 or − 5), *n* (%)	1009 (21.7)	933 (17.0)	0.11	< 0.01	< 0.01
Sedation adequacy, *n* (%)					
Insufficient	483 (8.9)	682 (10.4)	0.65	0.28	0.62
Adequate	4664 (85.4)	5324 (81.1)			
Excessive	313 (5.7)	555 (8.5)			

Variables are presented as absolute number (percentage of gathered observations). Comparisons made by multilevel mixed-effects Poisson regressions to simultaneously analyze the net effect of group assignment; the effect of time spent in ICU; the cumulative sedative effect, calculated by multiplying the group (enteral = 1, intravenous = 0) and the number of ICU staff shifts from group assignment, to highlight the adjunctive effects of the repeated sedative administration

*Abbreviations: IV* intravenous, *EN* enteral, *VNR* verbal numeric rating, *BPS* Behavioral Pain Scale, *RASS* Richmond Agitation Sedation Scale, *CAM-ICU* Confusion Assessment Method for Intensive Care Unit

There were minor differences between groups in clinical observations, and they arose only after considering the effects of time and group assignment together (Additional file 1: Table E5). The prevalence of sepsis was similar in the two groups, with no differences in the Sequential Organ Failure Assessment (SOFA) scores. Interestingly, even though this was not an outcome of the study, the EN sedation group received a larger amount of EN nutrition, both as planned calories - IV 22.6 (14.2–25) vs EN 23.6 (14.2–28.4) and as delivered calories - IV 22.3 (14.0–25.0) vs EN 22.8 (13.4–28.1) kcal/kg of ideal body weight, *p* < 0.01 for both comparisons.

## Discussion

This study compared two very different approaches for the management of agitation in critically ill patients, using different sedative drugs administered by the unusual EN route compared to the more common IV route. No real differences were found in the most important clinical outcomes.

In agreement with international guidelines [1, 4, 5], the target was a conscious patient for more than 80% of ICU days. This target was set more frequently in the EN group, but was achieved equally in both groups.

Among neurological indicators, studied throughout the ICU stay, the EN sedation protocol resulted in a similar incidence of delirium, while the RASS observed was slightly higher (Fig. 2b), which means a lower incidence of coma, but greater psychophysical agitation too. Indeed, the few self-removals of endotracheal tubes - about 5% among all participants - were more prevalent in the EN group. However, none of these caused death or serious complications, and the need to replace the tube was not different between groups, as reported elsewhere [15].

The feasibility of EN sedation was lower, as this strategy was associated with a higher incidence of protocol violations. It is impossible to say whether these violations were due to higher sedation targets being set by physicians, inadequate drug dosage, or timing of administration. This last point was frequently reported as a problem in centers not used to managing EN sedatives. The raw number of violations has to be considered together with the very different amounts of unplanned drug administered, at times reaching up to one third of the planned amounts for propofol. The absolute difference in the proportions of work shifts with violation was 19.2% in the EN group and 10.7% in the IV group. The EN group had a smaller number of violations in a larger number of patients (46.6% vs 4.2%), meaning that the reasons for violating the protocol were not the same throughout the ICU stay. Perhaps for this reason, nurses judged the EN sedation as being as adequate as IV sedation (89.7 vs 92.4%). From these figures, the separation between EN and IV sedation may seem an academic question and in many cases a combination of both might provide a more rational approach.

In managing psychophysical agitation, one must consider the pros and cons of physical and pharmacological means of contention (restraints and drugs) [32]. The cultural evolution [33] in the management of conscious critically ill patients involves greater consideration of their surveillance. An updated approach should integrate the MV mode and weaning process, body posture and physiotherapy, nutrition, and communication strategies - also involving relatives at the bedside in ICUs that are open to family visitors.

Interestingly, in the EN group there was a significant tendency to a lower impact on organ function: MV was more assisted than controlled, urinary output was higher, infection signs were weaker, gastrointestinal motility worked better (Additional file 1: Table E5).

The present study brings to light the need for clinical/cultural change [33] on two key points regarding the management of sedation therapy. First, despite great efforts to recommend aiming for the same RASS target in both arms, this decision was unexpectedly influenced by the group assignment (target RASS = 0 in 93.3% of the EN vs 82.9% of the IV group). Since this study is part of an educational research project, specific online medical education courses [34] were offered. All staff members were invited to increase their knowledge and to use validated tools to evaluate pain, sedation, and delirium. Moreover, since they had to simultaneously manage two different protocols, a phone counseling service from the coordinating center was always available. Despite this, different sedation targets remained, probably because of different knowledge and expertise in the use of the two protocols, requiring the titration of drugs with different pharmacokinetics. The “fear” of a lighter sedation target probably increased when IV drugs with a short half-life were used: since their effect could run out in a few minutes, the patient might become suddenly agitated. On the other side, a “fear of oversedation” due to accumulation of oral drugs could have played a role in targeting lighter sedation in the EN group.

Second, the habitual use of IV sedation led physicians to plan and administer smaller amounts of enteral nutrition, probably because they know its side effects on gastrointestinal motility. In this context, the challenge to accept less powerful drugs (like hydroxyzine) and to keep patients more awake might serve as a means for introducing good clinical practices.

The adequacy of nutrition and drugs administered through nasogastric/nasojejunal tubes strongly depends on the ICU staff teamwork and problem-solving attitudes. In order to obtain the best results with such EN drugs with slow onset and offset, we recommended starting with the highest doses in the first 24 h on ICU, to withdraw the IV drugs early. Thereafter, the drugs could be accurately titrated by using validated tools to measure the results, together with a constant effort to decrease/suspend the drugs as early as possible.

Hospital charges for the drugs are altogether very low in relation to other ICU costs. The charges for planned sedatives were lower in the EN group and were higher for unplanned drugs. Considering the costs for neuroactive drugs altogether, there was a significant difference (IV 4.15 vs EN 2.39 €/MV day), meaning both that charges for antipsychotics were not increased, and charges for analgesics were slightly lower, probably because of melatonin’s pain-relieving effect [35]. These charges are much lower than those reported in the literature; hospital costs could be significantly higher with respect to new drugs and approaches, like dexmedetomidine or sevoflurane [25].

### Study limitations and strengths

The unexpected difference in RASS targets was a significant limitation of this study, which might have favored the IV sedation protocol: as the patients were wanted to be more sedated, reaching such a target was easier. There are also several other limitations, like the single-blind design of the study, the data recording by clinical staff, the lack of anamnestic data on alcohol or substance abuse, the non-protocolized weaning from MV, the lack of a long-term cognitive outcome evaluation, and the sedation assessment over a whole nursing shift (prevalent RASS), which is very subjective. Moreover, some clinical practices were being introduced for the first time (EN protocol, conscious target, use of validated tools for neurological monitoring) in a substantial proportion of centers when the study was started. Data were gathered some years ago, and different skills and habits among intensivists in the use of sedatives could have played some role, particularly in the use of benzodiazepines, which is discouraged nowadays. Last, half the patients in the EN group had protocol violations, meaning the groups were not adequately separated.

The strengths of the present study are its design coherent with guidelines, always suggesting an early conscious sedation target [1]. Rather than making it different in the two groups [31, 36], two separate strategies were compared in the achievement of the same shared goal: a calm, conscious, and cooperative critically ill patient. Moreover, the lack of homogeneity among participant centers could render the results generalizable. Even with the large number of violations, the two strategies seem to be comparable: non-skilled centers can immediately use the EN strategy too.

## Conclusions

The EN protocol for the management of sedation in high-risk critically ill patients was not associated with any improvement in the rate of achievement of the desired level of sedation. Some hypothesis-generating advantages, like the light sedation target or the lower costs, might reflect a cultural change regarding the EN route. The use of this route for “gentle patient sedation” appeared possible and safe: when aiming at the target of a conscious critically ill patient, this unusual approach - based on drugs with weaker and longer effect - does appear to offer some benefits.

## Additional file

Additional file 1: Table E1 Description of participating ICUs. Table E2 Multivariate generalized linear model of main outcome. Table E3 Reasons for protocol violation. Table E4 Neuroactive drug doses and hospital charges. Table E5 Daily clinical measurements. Figure E1 Kaplan–Meier plot for ICU survival estimates. (DOC 3129 kb)

## Abbreviations

BMI: Body mass index
BPS: Behavioral Pain Scale
CAM-ICU: Confusion Assessment Method for ICU
COPD: Chronic obstructive pulmonary disease
EN: Enteral
ET: Endotracheal
ICU: Intensive care unit
IQR: Interquartile range
IV: Intravenous
MV: Mechanical ventilation
RASS: Richmond Agitation Sedation Scale
SAPS: Simplified Acute Physiology Score
SedaEN: Enteral versus intravenous sedation trial
SOFA: Sequential Organ Failure Assessment
VNR: Verbal numeric rating
