# Supplementary material for: Enteral versus intravenous approach for the sedation of critically ill patients: a randomized and controlled trial
Source: Crit Care. 2019 Jan 7;23:3. doi: 10.1186/s13054-018-2280-x (PMC6323792; doi:10.1186/s13054-018-2280-x)

**Enteral versus intravenous approach for the sedation of critically ill patients**

Giovanni Mistraletti^1,2^, Michele Umbrello^2^, Silvia Salini^3^, Paolo Cadringher^4^, Paolo Formenti^2^, Davide Chiumello^2,5^, Cristina Villa^1^, Riccarda Russo^4^, Silvia Francesconi^6^, Federico Valdambrini^7^, Giacomo Bellani^8^, Alessandra Palo^9^, Francesca Riccardi^10^, Enrica Ferretti^11^, Maurilio Festa^12^, Anna Maria Gado^13^, Martina Taverna^14^, Cristina Pinna^15^, Alessandro Barbiero^3^, Pier Alda Ferrari^3^, and Gaetano Iapichino^1,2^, and the SedaEN investigators.

^1^ Dipartimento di Fisiopatologia Medico-Chirurgica e dei Trapianti, Università degli Studi di Milano.

^2^ SC Anestesia e Rianimazione, ASST Santi Paolo e Carlo, Ospedale San Paolo – Polo Universitario, Milano.

^3^ Dipartimento di Economia, Management e Metodi quantitativi, Università degli Studi di Milano.

^4^ UOC Anestesia e Terapia Intensiva, Fondazione IRCCS Ca' Granda, Ospedale Maggiore Policlinico, Milano.

^5^ Dipartimento di Scienze della Salute, Università degli Studi di Milano.

^6^ UOC Anestesia e Rianimazione, ASST Monza, Ospedale di Desio (MI).

^7^ UO Anestesia e Rianimazione, ASST Ovest Milanese, Ospedale Nuovo di Legnano (MI).

^8^ Dipartimento di Medicina e Chirurgia, Università degli Studi Milano Bicocca, A.O. San Gerardo, Monza (MI);

^9^ Dipartimento Medicina Intensiva, IRCCS Fondazione Policlinico San Matteo, Pavia.

^10^ UO Anestesia e Rianimazione 2, IRCCS San Matteo, Pavia.

^11^ SC Anestesia Rianimazione B DEA, Ospedale San Giovanni Bosco, Torino.

^12^ SCDU Anestesia e Rianimazione, AOU San Luigi Gonzaga di Orbassano (TO).

^13^ UO Anestesia e Rianimazione, AO Cardinal Massaia, Asti.

^14^ UO Anestesia e Rianimazione, AO Santi Antonio e Biagio e Cesare Arrigo, Alessandria.

^15^ UO Anestesia e Rianimazione, Dipartimento di Area Critica, Nuovo Ospedale Civile Sant’Agostino Estense, Modena.

Electronic supplementary material


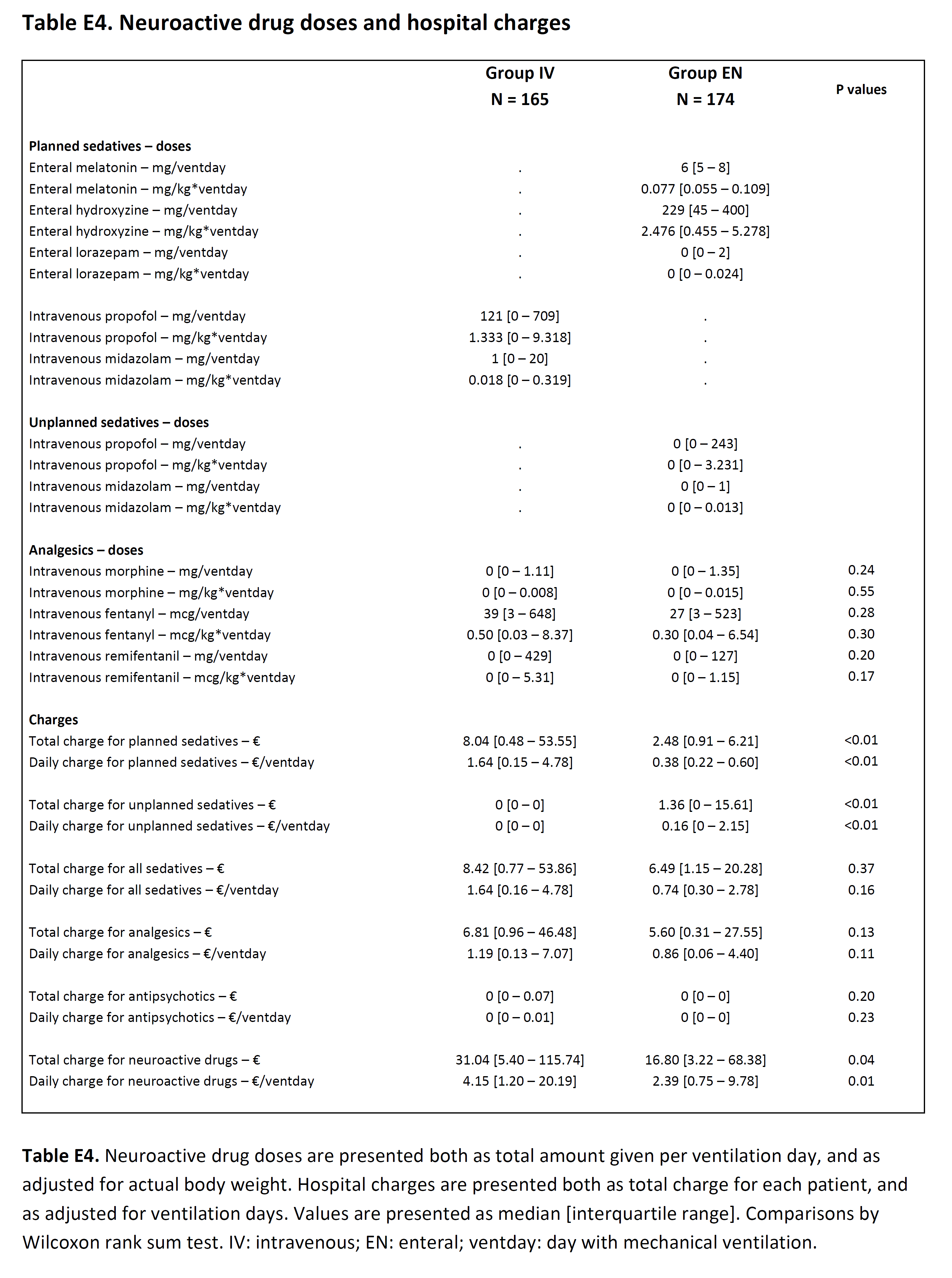

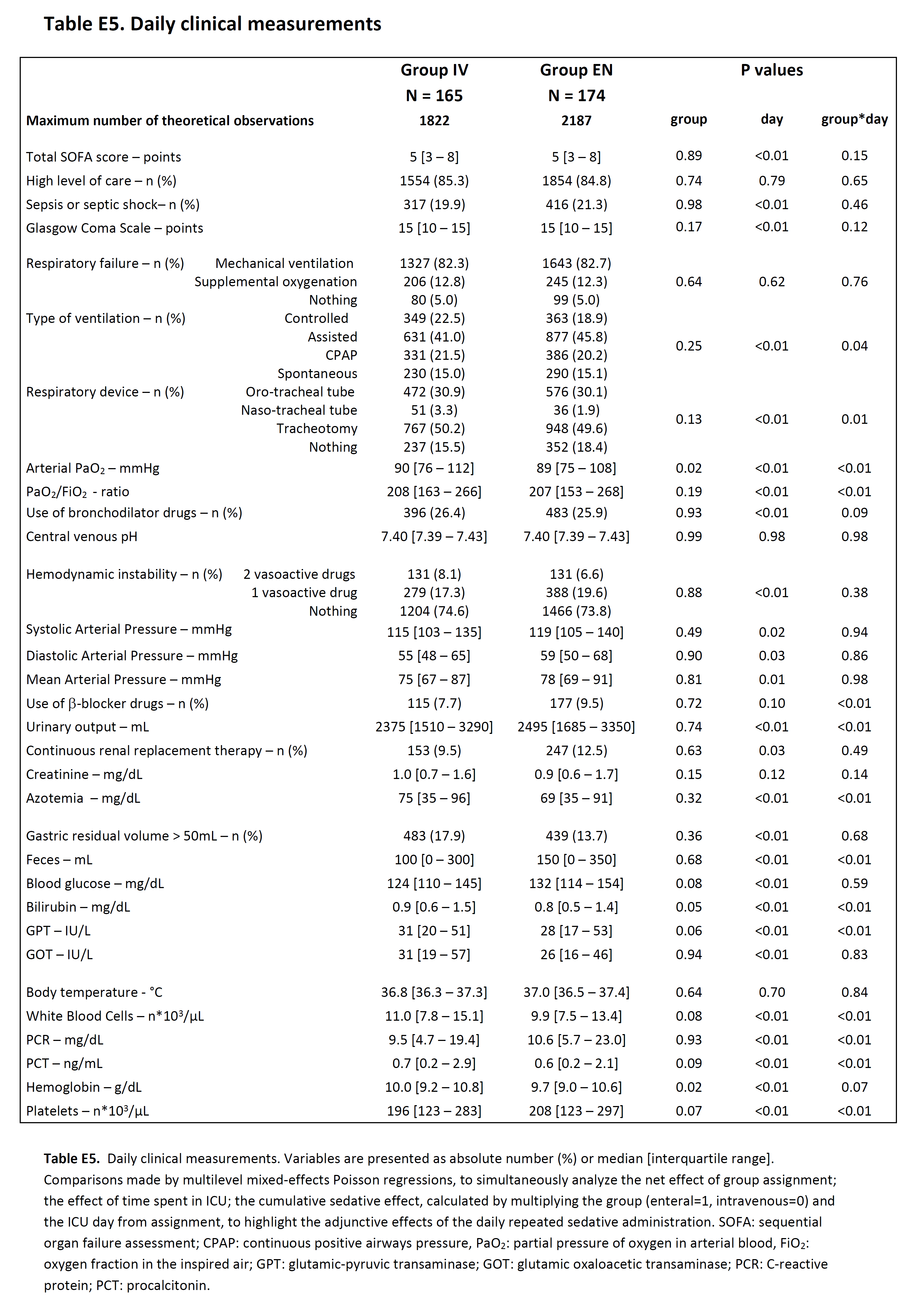

Supplement: Supplementary file 1 — Table E1 Description of participating ICUs. Table E2 Multivariate generalized linear model of main outcome. Table E3 Reasons for protocol violation. Table E4 Neuroactive drug doses and hospital charges. Table E5 Daily clinical measurements. Figure E1 Kaplan–Meier plot for ICU survival estimates. (DOC 3129 kb) [file 13054_2018_2280_MOESM1_ESM.doc]
